# Factors Influencing Graft Outcomes Following Diagnosis of Polyomavirus –Associated Nephropathy after Renal Transplantation

**DOI:** 10.1371/journal.pone.0142460

**Published:** 2015-11-06

**Authors:** Gang Huang, Lin-wei Wu, Shi-Cong Yang, Ji-guang Fei, Su-xiong Deng, Jun Li, Guo-dong Chen, Qian Fu, Rong-hai Deng, Jiang Qiu, Chang-xi Wang, Li-zhong Chen

**Affiliations:** 1 Department of Organ Transplantation, The First Affiliated Hospital, Sun Yat-sen University, Guangzhou, Guangdong, China; 2 Department of Pathology, The First Affiliated Hospital, Sun Yat-sen University, Guangzhou, Guangdong, China; University of Toledo, UNITED STATES

## Abstract

**Background:**

Polyomavirus associated nephropathy (PVAN) is a significant cause of early allograft loss and the course is difficult to predict. The aim of this study is to identify factors influencing outcome for PVAN.

**Methods:**

Between 2006 and 2014, we diagnosed PVAN in 48 (7.8%) of 615 patients monitored for BK virus every 1–4 weeks after modification of maintenance immunosuppression. Logistic or Cox regression analysis were performed to determine which risk factors independently affected clinical outcome and graft loss respectively.

**Results:**

After 32.1±26.4 months follow-up, the frequencies of any graft functional decline at 1 year post-diagnosis, graft loss and any graft functional decline at the last available follow-up were 27.1% (13/48), 25.0% (12/48), and 33.3% (16/48), respectively. The 1, 3, 5 year graft survival rates were 100%, 80.5% and 69.1%, respectively. The mean level of serum creatinine at 1 year post-diagnosis and long-term graft survival rates were the worst in class C (p<0.05). Thirty-eight of 46 (82.6%) BKV DNAuria patients reduced viral load by 90% with a median time of 2.75 months (range, 0.25–34.0 months) and showed better graft survival rates than the 8 patients (17.4%) without viral load reduction (p<0.001). Multivariate logistic regression analysis showed that extensive interstitial inflammation (OR 20.2, p = 0.042) and delayed fall in urinary viral load (>2.75 months for >90% decrease) in urine (OR 16.7, p = 0.055) correlated with worse creatinine at 1 year post-diagnosis. Multivariate Cox regression analysis showed that extensive interstitial inflammation (HR 46988, p = 0.032) at diagnosis, and high PVAN stage (HR 162.2, p = 0.021) were associated with worse long-term graft survival rates.

**Conclusions:**

The extent of interstitial inflammation influences short and long-term graft outcomes in patients with PVAN. The degree of PVAN, rate of reduction in viral load, and viral clearance also can be used as prognostic markers in PVAN.

## Introduction

The human BK polyomavirus (BKV) can infect the majority of the population and subsequently remains dormant in the kidney without consequence. However, under conditions of immunosuppression, especially renal transplantation, reactivation, and replication, may occur, causing an interstitial nephritis in the renal allograft. Polyomavirus-associated nephropathy (PVAN) was first diagnosed in Pittsburgh in 1993 by Dr. Randhawa in a renal transplant recipient suspected of having acute rejection [1]. It has emerged as the most common infectious disease in the kidney allograft with an incidence of 2% to 10% [2]. PVAN progressively affects graft function and increases the risk of graft loss from <10% to more than 90% [3–6].

Given the small number of published interventional studies, the clinician is often faced with uncertainty in predicting the clinical outcome of the graft. Clinical factors reported to be associated with worse prognosis include deceased donor, female recipient, high serum creatinine at diagnosis, late diagnosis, and plasma peak viral load [7–9].

Although the biopsy findings at diagnosis are proposed to be a predictive tool for assessing prognosis [10, 11], the rate of BKV viral load reduction and clearance after modification of maintenance immunosuppression have generally not been predictive of outcome. To date, few studies have evaluated both the kinetics of BKV viral load and clinical variables to predict the outcome.

In the current investigation, we used quantitative PCR for BKV DNA load in urine and plasma and quantitative urine cytology to evaluate BKV infection in kidney transplant (KTx) recipients who received renal graft biopsies concurrently to identify PVAN. Moreover, we followed up PVAN patients after modification of maintenance immunosuppression to observe the clinical course hoping to identify prognostic variables of PVAN.

## Materials and Methods

### Patient selection

From March 2006 to August 2014, 615 renal transplant recipients at our institution who underwent an allograft biopsy with an immunohistochemistry assay for polyomavirus were screened for BKV reactivation concomitantly, which consisted of urine cytological evaluation and quantitative PCR of both urine and plasma for BKV DNA. Forty-eight kidney transplant (KTx) recipients diagnosed with definitive PVAN were included in this study.

### Ethical statement

Study approval was obtained from the Ethics Committee of the First Affiliated Hospital, Sun Yat-sen University, Guangzhou, China. All patients gave their written informed consent to participate in the study, which was conducted in accordance with the Helsinki Declaration.

### BKV monitoring

#### Urinary cytologic studies

Urinary cytology smears were stained by the Papanicolaou method and evaluated for the presence of cells with intranuclear viral inclusions (decoy cells), which were counted [number per 10 high-power fields] as described elsewhere [12–14].

#### Virologic studies

Quantification of the urine and plasma BKV load were performed by quantitative PCR (Q-PCR) (MJ Research, Waltham, MA, USA). Specimen collection and processing, PCR primers, TaqMan probe, plasmid standard containing the targeted BKV *VP1* gene, amplification protocols, PCR precautions, and quality assurance have been described elsewhere [12,13]. Urine or plasma BKV load was expressed in BKV genome copies per milliliter of urine or plasma. The lower limit of quantitation was 1000 copies per milliliter.

#### Diagnosis of PVAN

Hematoxylin & Eosin, Periodic acid Schiff, and Masson’ silver trichrome stains were performed on all biopsies. PVAN was defined by the typical cytopathic effect and confirmed by positive immunohistochemical nuclear staining with anti-SV40 large T antigen monoclonal antibody as previously described [11–15]. The histologic features of PVAN were classified using the American Society of Transplantation (AST) schema and assigned to PVAN categories -A, B, and C based on the guidelines of Hirsch et al. [16]. Histologic viral load was assessed semi-quantitatively as the percentage of tubules that stained positive for BKV using a 4 tier system (<10%, 10–25%, 25–50%, and >50%) [3]. Histologic lesions were scored using the Banff 1997 schema of renal allograft pathology. Acute rejection (AR) was defined by the Banff criteria [17].

A total volume of 600mL urine was centrifuged at 1500g for 15min to collect the tubular cells. An average of 3–6 grids per patient were examined to detect the viral particles in the tubular cells using an electron microscopy (Philips, Eindhoven, The Netherland). Cells had viral particle (with a diameter of about 45nm) detection in cyptoplasm or nucleus will be regarded as infected cells. At least 2–10 cells will be examined per grid before declaring that none viral particle is detected.

### Modification of maintenance immunosuppression and follow-up

Two strategies for reducing the immunosuppressive load were considered for the PVAN patients but without acute rejection (AR) and included switching from tacrolimus (Tac) to cyclosporine A (CsA), and reducing calcineurin inhibitors by 75~50% of the original level. Further reduction of immunosuppression was guided by the whole blood trough level (Tac trough level ≤5 ng/ml, CsA trough level ≤120 ng/ml). Mycophenolic acid (MPA) was reduced or maintained the original level [mycophenolate mofetil (MMF) ≤1 g/d; mycophenolate sodium≤0.72 g/d]. Prednisone was not changed.

All patients were followed for at least 1 years post-diagnosis with regular monitoring of serum creatinine (Scr), trough Tac/CsA levels, and BKV replication every week for the first month followed by 2~4 weeks for next 3 months and then every 3–6 months.

Delayed graft function (DGF) was defined as the need for dialysis within the first week after transplantation, except when only one session was performed immediately after surgery due to hyperkalemia or fluid overload. Severe pneumonia was defined as pneumonia occurring in the first year accompanied with acute respiratory distress syndrome.

### Assessment of 1-year and long-term outcome of PVAN after modification of maintenance immunosuppression

The mean graft follow-up period of BKVN patients after diagnosis was 32.1±26.4 months. The study endpoint was date of return to dialysis, or most recent clinical record. The following adverse outcomes were assessed for each patient: (a) Graft loss: patient death or return to dialysis. (b) Any graft functional decline: Graft loss or sustained increase in Scr >30% compared to the value at PVAN diagnosis.

### Statistical analysis

Analysis was performed using SPSS software (version 13.0, SPSS Inc., Chicago, IL, USA). Continuous variables were expressed as mean ± standard deviation (SD) or as median (minimal–maximal range) and compared using the t-test or Wilcoxon signed-rank test. Pearson’s chi-square test, Fisher’s exact test, and the Mann-Whitney-U test were used to analyze the data, as appropriate.

The 1, 3, 5 year-graft survival rates rate were calculated by Kaplan-Meier curves and compared among groups by Log rank test.

Univariate logistic and Cox regression analysis were performed to determine which risk factors affected clinical outcome and graft loss respectively. After univariate regression, recipient, transplant characteristics, and specific variables associated with p-values <0.05 by univariate analysis were entered into multivariate analysis to determine which factor affected graft outcome independently. Results were expressed as odds ratios (OR) with respective 95% confidence intervals. Cox’s proportional hazard regression analysis was used to assign relative risk (RR) to factors predictive of graft loss. A P-value <0.05 was considered significant.

## Results

### Population characteristics

Demographic information and baseline clinical data for the 48 recipients diagnosed as PVAN according to the pathologic classification are summarized in Table 1. The PVAN was class A, B, and C in11, 31, and 6 patients, respectively. Of note, the predominant oral immunosuppressive regimen was Tac, MPA and prednisone (95.8%). CsA, MPA and prednisone was administered in only 2 patients (4.2%). The mean interval between KTx and diagnosis of PVAN was 14.7±10.4 months. A biopsy had been performed for BKV surveillance (without a rise of the Scr) in 9 (81.8%) patients with class A PVAN. This incidence was higher than that observed for PVAN class B and C (p<0.001). Scr was lower in class A PVAN (164.6±36.1 umol/L) compared to class B (181.9±58.1 umol/L) and C (369.7±158.7 umol/L) (p<0.001) (Table 2). On the day of diagnosis of PVAN, patients with class B disease were characterized by the highest median level of decoy cell [43/10 HPF, range (0-125/10 HPF)], urine BK viral load [6.00×10^9^ copies/ml, range (8.10×10^5^−1.20×10^12^copies/ml)], and plasma BK viral load [5.34 ×10^4^ copies/ml, (0–5.20×10^6^copies/ml)], compared to class A and C PVAN (Table 1). The distribution of i, ct, and ci scores was significantly different among class A, B, and C PVAN (all P<0.05).

**Table 1:** Demographic and Clinical Characteristics of 48 PVAN Patients according to the Pathologic Class.

	Class A (n = 11)	Class B (n = 31)	Class C (n = 6)	P value
Age at transplant	36±7	39±12	41±13	0.609
Male gender [n(%)]	10(90.9)	15(48.4)	4(66.7)	0.044
Living donor[n(%)]	3(27.3)	6(19.4)	1(16.7)	0.827
**Immunosuppressive Regimen**				
Anti-IL-2R [n(%)]	7(63.6)	9(29.0)	3(50.0)	0.112
ATG [n(%)]	7(63.6)	22(71.0)	2(33.3)	0.210
Tac -MPA[n(%)]	10(90.9)	30(96.8)	6(100)	0.607
**Complications**				
AR before PVAN[n(%)]	3(27.3)	10(32.3)	4(66.7)	0.221
DGF[n(%)]	0(0)	3(9.7)	0(0)	0.416
Severe pneumonia [n(%)]	2(18.2)	5(16.1)	3(50.0)	0.169
**Diagnosis of PVAN**				
Time posttransplant (m)	11±6	16±12	15±3	0.376
Biopsy performed for BKV surveillance	9(81.8)	7(22.6)	0(0)	<0.001
Decoy cells(/10 HPF)	23(2–85)	43(0–125)	17(0–35)	0.015
BKV DNAuria (copies /ml)	1.38 ×10^7^(1.00×10^5^−9.00×10^10^)	6.00×10^9^(8.10×10^5^−1.20×10^12^)	4.92×10^8^(0–6.00×10^10^)	0.030
BK viremia(copies /ml)	1.60 ×10^4^(0–1.22×10^5^)	5.34 ×10^4^(0–5.20×10^6^)	8.49×10^2^(0–2.25×10^5^)	0.079
Histologic finding at diagnosis				
cy score	1.09±0.3	1.71±0.9	1.67±0.5	0.065
i score	0.18±0.4	1.61±0.5	2.50±0.5	<0.001
ct score	0.36±0.7	1.48±0.6	2.33±0.8	<0.001
ci score	0.18±0.4	1.29±0.7	2.50±0.6	<0.001

Anti-IL-2R, monoclonal antibody to the IL-2 receptor; ATG, Anti-thymocyte globulin

**Table 2:** 1-year and Long-term Graft Outcome, BKV Reduction and Clearance of PVAN Classified as A, B or C after Modification of Maintenance Immunosuppression.

	A (n = 11)	B (n = 31)	C (n = 6)	P value
**Graft outcome**				
Scr at diagnosis (umol/L)	164.6±36.1	181.9±58.1	369.7±158.7	<0.001
Graft loss 1 year after diagnosis[n(%)]	0(0)	2(6.5)	4(66.7)	<0.001
Any graft functional decline 1 year after diagnosis [n(%)]	1(9.1)	8(25.8)	4(66.7)	0.037
Scr 1 year after diagnosis(umol/L)	165.5±85.3	203.0±108.8	363.0±84.9	0.052
Graft loss at the latest follow-up[n(%)]	0(0)	7(22.6)	5(83.3)	0.001
Any graft functional decline at the latest follow-up [n(%)]	1(9.1)	10(32.3)	5(83.3)	0.008
Scr at the latest follow-up(umol/L)	168.2±88.3	177.5±95.0	486.0±0	0.009
**Reduction of BKV replication by 90% [n(%)]**				
BKV DNAuria	10/11(90.9)	26/31(83.9)	2/4(50.0)	0.002
BK viremia	9/10 (90.0)	25/28 (89.3)	1/3(33.3)	0.024
**Clearance of BKV replication [n(%)]**				
Decoy cells	10/11(90.9)	17/30 (56.7)	2/5(40.0)	0.070
BKV DNAuria	7/11 (63.6)	5/31 (16.1)	1/4(25.0)	<0.001
BK viremia	9/10(90.0)	23/28(82.1)	1/3(33.3)	0.039

### 1-year and long-term outcome of PVAN after modification of maintenance immunosuppression

**Fig 1:**
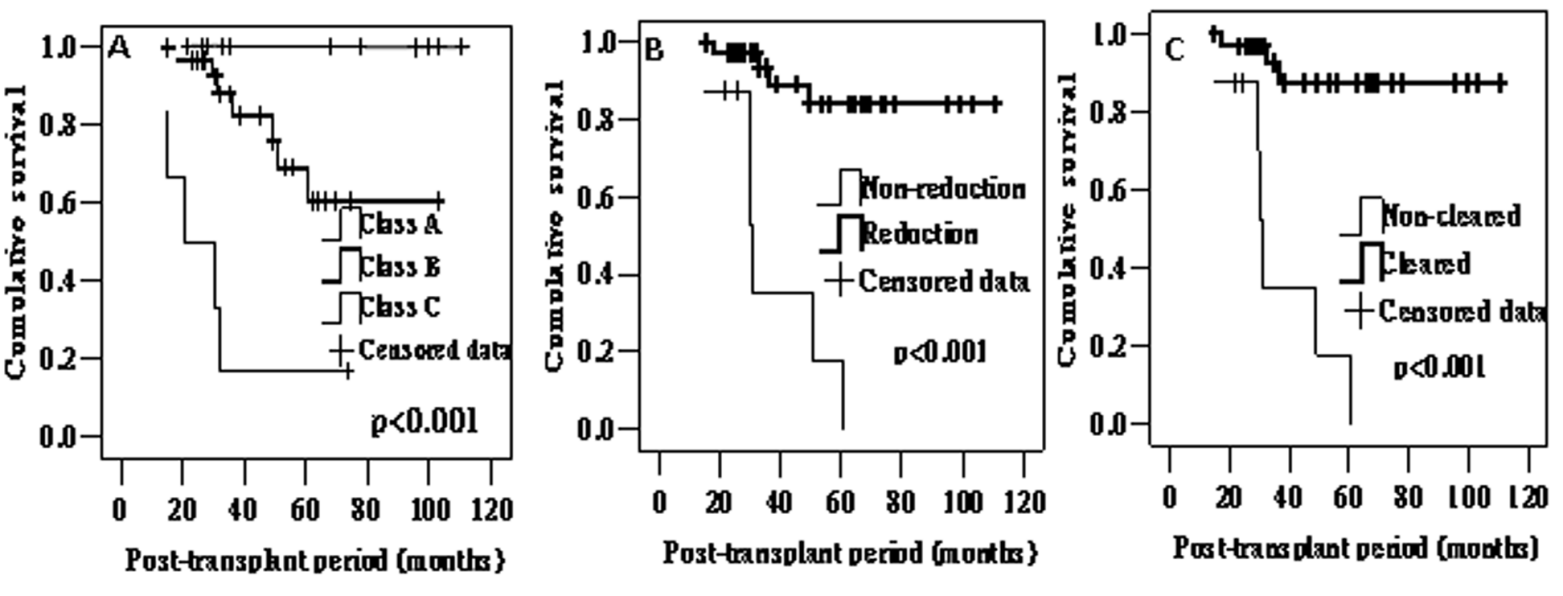
Kaplan-Meier estimates of long-term graft outcome in patients of PVAN. Patients of PVAN were classified as A, B or C (A), and with and without reduction of BKV DNAuria by 90% (B) and clearance of viremia (C).

After modification of maintenance immunosuppression, graft loss and any graft functional decline at 1 year occurred in 6 (12.5%) and 13 (27.1%) patients. For those (n = 42) patients with a functioning graft at 1 year post-diagnosis, the mean level of Scr at 1 years was 200.5 ± 108.1μmol/L (range 90–531μmol/L). At last available follow-up, the frequencies of graft loss and any functional decline were 25% (12/48) and 33.3% (16/48) respectively. The 1, 3, 5 year graft survival rates rates were 100%, 80.5% and 69.1%, respectively. The frequency of graft loss, any functional decline, and the mean level of Scr at 1 year post-diagnosis and last available follow-up was higher in class C PVAN compared to class A and B (Table 2). Long-term graft outcome evaluated was the worst in class C PVAN (Fig 1A).

### Effect of modification of maintenance immunosuppression and time of 90% reduction of BKV load, and viral clearance in PVAN patients

More patients with PVAN class A had reduction of BKV replication by 90% and cleared BKV after modification of maintenance immunosuppression (Table 2). There were statistically significant differences in the rate of reduction and clearance of BKV DNAuria and viremia among the three groups (p<0.05).

Thirty-three of 41 (80.5%) BK viremia patients with clearance of viremia on follow-up showed better graft survival rates than the 8 patients (19.5%) without viral clearance (p<0.001, Fig 1B). The median time to clearance of viremia was 3.5 months (range, 0.25∼32.0 months). Graft survival rates in patients with and without clearance of viremia was 87.3% and 35.0% respectively at 3 years. The corresponding at five years post-transplant graft survival rates were 87.3% and 17.5%.

Most patients (33/46, 71.7%) continued to excrete virus in urine for 25.6 ± 25.5 months with a median viral load of 4.42x10^6^ copies/ml (IQR, 3.55x10^5^∼5.73x10^7^), and clearance of BKV DNAuria as well as viremia could be documented in only in 10 cases. Thirty-eight of 46 (82.6%) BK BKV DNAuria patients with 90% reduction of BKV load on follow-up showed better graft survival rates (p<0.001, Fig 1C). The median time to 90% reduction of BKV load was 2.75 months (range, 0.25∼34 months).

Presuming an exponential multiplication of viral load during infection, a significant viral load reduction was defined as a reduction of at least 90% (1 log is 10 fold) [18]; the development of viral load reduction was studied by measuring the time (months) of quantitative PCR decline by 90% [18]. According to the median time for viral load reduction by 90% and clearance in urine and plasma, this cutoff was chosen to distinguish between rapid and slow reduction and clearance of BKV DNAuria and viremia (Table 3). Because the median time to clearance of BKV DNAuria was 22 months, which was longer than the minimum follow-up time of all the patients (12 months), the clearance of BKV DNAuria was not divided into rapid and slow groups.

**Table 3:** The Median Time to Reduction and Clearance of BKV DNAuria and Viremia.

	Reduction of BKV replication by 90%			Clearance of BKV replication		
	Median time (month)	Rapid	Slow	Median time (month)	Rapid	Slow
BKV DNAuria	2.75(0.25–34)	≤2.75(n = 20)	>2.75(n = 26)	22.0(0.25–48.0)	-	-
BK viremia	2.0(0.25–30)	≤2.0 (n = 18)	>2.0 (n = 23)	3.5(0.25–32)	≤3.5 (n = 17)	>3.5 (n = 24)

### Determinants of 1-year graft function

Logistic regression analyses of different clinical parameters on any graft functional decline at 1 year post-diagnosis are shown in Table 4. On univariate analysis, high level of Scr at the time of biopsy, high degree of PVAN classification, and high grade of i, ci, ct score on biopsy were significantly associated with worse allograft function at 12 months post biopsy. Conversely, reduction or clearance of viremia, and rapid reduction of BKV DNAuria were associated with better preservation of graft function. The factors that did not influence outcome included patient characteristics (age, gender, living donor), immunosuppressive regimen (IL-2 receptor blocker, Thymoglobulin, Tac -MPA), complications post-transplant (AR, DGF, Severe pneumonia), characteristics at diagnosis of PVAN (interval between KTx and diagnosis of PVAN, BKV surveillance biopsy, urinary and plasma BKV load, urinary decoy cell number at the time of biopsy), viral response to modification of maintenance immunosuppression (reduction and clearance of BKV DNAuria, time of viremia clearance and reduction by 90%), and histologic score of viral load at diagnosis (cy score).

**Table 4:** Analysis of factors associated with any graft functional decline 12 months after the diagnosis and long-term graft loss in PVAN patients.

	Univariate analysis^a^		Multivariate analysis	
	OR& RR (95% CI)	p value	OR&RR (95% CI)	p value
**Any graft functional decline 12 months after the diagnosis**				
Scr at diagnosis	1.01(1.00–1.02)	0.028	1.01(1.00–1.03)	0.194
Reduction of viremia by 90%	0.037(0.005–0.297)	0.002	8.74(0.06–1240.53)	0.391
Clearance of viremia	0.044(0.007–0.290)	0.001	0.01(0.00–1.62)	0.077
Rapid reduction of BK DNAuria	0.139(0.025–0.762)	0.023	0.06(0.003–1.06)	0.055
Pathological parameters				
Classification of PVAN	4.68(1.21–18.17)	0.026	0.44(0.02–11.62)	0.622
i score	7.15(1.73–29.56)	0.007	20.16(1.11–365.57)	0.042
ci score	2.41(1.09–5.34)	0.030	4.48(0.18–114.85)	0.365
ct score	2.42(1.02–5.73)	0.045	0.04(0–3.49)	0.157
**Long-term graft loss**				
Scr at diagnosis	1.01(1.00–1.01)	0.008	0.99(0.96–1.01)	0.262
BKV replication				
Clearance of viremia	0.08(0.02–0.29)	0.000	2.91(0.02–445.86)	0.677
Reduction of BKV DNAuriA by 90%	0.09(0.03–0.30)	0.000	0.007(0–1.39)	0.066
Reduction of viremia by 90%	0.10(0.03–0.35)	0.000	0.073(0.001–5.35)	0.232
Rapid clearance of of viremia	0.15(0.03–0.73)	0.018	0.01(0–2×10^126^)	0.962
Rapid reduction of of viremia	0.19(0.04–0.88)	0.034	570.28(0–2×10^132^)	0.967
Pathological parameters				
Classification(A/B/C))	7.36(2.61–20.76)	0.000	162.16(2.12–12385.55)	0.021
cy score	2.28(1.23–4.25)	0.009	9.63(0.72–128.83)	0.087
i score	49.73(5.54–446.9)	0.000	46988.14(2.49–9×10^8^)	0.032
ci score	3.23(1.39–7.47)	0.006	2.18(0.11–43.70)	0.611
ct score	3.56(1.36–9.32)	0.010	0.076(0.001–4.18)	0.207

^a^Logistic and Cox regression analysis were performed to determine which risk factors affected any graft functional decline 12 months after the diagnosis and long-term graft loss respectively. Results were expressed as odds ratios (OR) or relative risk (RR) with respective 95% confidence intervals.

On multivariate logistic regression analysis only severe interstitial inflammation (OR = 20.16; 95%CI: 1.11–365.57) was as an independent predictor of poor graft function at 12 months post-diagnosis (p = 0.042). Rapid reduction of BKV DNAuria and clearance of viremia were associated with a trend towards improvement of graft function (p = 0.055, and 0.077, respectively).

### Determinants of long-term graft outcome

On univariate analyses using Cox’s regression model (Table 4), factors associated with graft loss were high level of Scr at diagnosis, high degree of PVAN classification, and high grade of cy, i, ci, ct score on biopsy. Conversely, clearance of viremia, reduction of BKV DNAuria and viremia, and rapid reduction and clearance of viremia, were associated with better long-term graft outcome. Other factors mentioned above were not associated with the long-term graft outcome (Table 4). On multivariate analysis, PVAN classification and i score remained significant. Reduction of BKV DNAuria was associated with a trend towards better long-term graft outcome (p = 0.066). Intensive BKV positive tubules was associated with a trend towards graft loss (p = 0.087).

## Discussion

This study reveals factors associated with graft functional decline and long-term outcome in KTx recipients with PVAN. These potential factors include patient characteristics, immunosuppressive regimen, complications posttransplant, characteristics at diagnosis of PVAN, viral response to modification of maintenance immunosuppression, and histologic findings at diagnosis.

Based on histologic classification, there are some characteristics in every PVAN class at diagnosis, which have the potential to influence graft outcome. The majority of patients in class A (9/11) received graft biopsy for BKV surveillance and not for a rise of the Scr. This was less frequently the case for class B (7/31) and class C (0/6). Patients with class B were characterized by the highest BKV load at diagnosis. The mean level of Scr, and scores for cy, i, ci, and ct were the highest in the patients with class C. However, except the histologic lesions, other factors of baseline did not influence graft outcome independently in our study, which is not consistent with some previous reports. For example, Masutani et al [19] reported that patients with plasma viral load in the highest quartile at time of diagnosis of BKVN tended to have worse 5 year survival (25.0%) compared to patients in the lowest quartile, graft survival rates (56.3%). Other clinical factors reported to be associated with worse prognosis include deceased donor, male recipient, high Scr at diagnosis, and late diagnosis [5–7].

Although with early diagnosis the majority of patients can respond favorably, approximately 15% of still progress to graft loss. The actual consequence of the disease is more serious since 38% of patients continue to show decline in graft function, while 24% show a major decline characterized by sustained increase in serum creatinine of ≥ 50% compared to the value at the time of PVAN diagnosis. The data above is based on a 20±11 months follow-up study [5], which is similar with ours except for longer follow-up period. The frequency of total graft loss, and the mean level of Scr at 1 year post-diagnosis and last available follow-up in the patients with class A was better than those in the patients of class B and C (Table 2). Long-term graft outcome evaluated was the worst in class C (Fig 1A).

On multivariate Cox’s regression analysis, histological classification was an independent factor for long-term graft outcome due to advanced interstitial fibrosis and tubular atrophy in class C at diagnosis. Our data indicate that definitive PVAN can still occur with stable allograft function and only detectable by surveillance biopsies. 81.8% patients in class A fall into this category. Detection of PVAN in the early stage is very likely the key step for the success of immunosuppression reduction strategies. Indeed, all studies—including ours—that report favorable outcomes of immunosuppression reduction, detected BKV-viremia early posttransplant allowing for timely therapeutic intervention [5, 20–22]. However, if the diagnosis of definitive PVAN is made at a later time point (e.g. class C), reduction of immunosuppression is less successful, most likely due to more advanced irreversible damage of the allograft, which lead to rates of graft loss >80% [17, 23].

A composite system to classify the disease based on viral cytopathic effect, extent of interstitial inflammation, tubular atrophy, and severity of interstitial fibrosis were also involved in the analysis. Extensive interstitial inflammation can influence adversely on both graft function at 1 year post-diagnosis and long-term graft survival rates. This is in agreement with Randhawa et al. [19], who demonstrated that advanced interstitial inflammation at diagnosis correlated with poorer graft outcome. I inflammatory lesions in biopsies with PVAN may also reflect ongoing diseases like antibody mediated rejection, T-cell mediated rejection, and glomerulonephritis [24].

If the frequency of viral reduction and clearance is introduced to the analysis, thirty-three of 41 (80.5%) BK viremia patients with clearance of viremia on follow-up showed better graft survival rates than the 8 patients (19.5%) without viral clearance (p<0.001, Fig 1). This is in line with Prof. Randhawa’s report [19], which showed 81.3% (26/32) patients with clearance of viremia on follow-up showed better graft survival rates than the 18.7% patients (6/32) without viral clearance. Furthermore in our study, most patients (33/46, 71.7%) continued to excrete virus in the urine. However, thirty-eight of 46 (82.6%) BKV DNAuria patients reduced virus in urine by 90%, in whom better graft survival rates can also be observed. Multivariate Cox’s regression analysis also showed that viral reduction by 90% in urine was associated with a trend towards better long-term graft outcome independently.

According to the mathematical model about BKV replication developed by Funk et al [25], optimal interventions should curtail BKV replication by ≥90% and can be identified by the rapid and almost parallel decline of plasma and urine BKV loads within <10 weeks. Although, the viral reduction by 90% in plasma and urine occurred in most of the patients, the time from modification of maintenance immunosuppression to the reduction was very different individually. On multivariate logistic regression analysis, rapid reduction of BKV DNAuria was associated with a trend towards improvement of graft function at 1 year post-diagnosis (p = 0.055). This is in agreement with the report by Schwartz et al [18] that patients clearing the virus quickly showed more often a stable or increasing eGFR, compared with those clearing it slowly. In that report, rapid viral reduction in the PVAN patients was associated with stable or increasing GFR (84%) compared with slow viral reduction (33%; P = .0004). It should be emphasized that rate of reduction of BKV DNAuria will determine whether decline of graft function will occur early after diagnosis. This indicates long-persisting BKV infection and failure to mount BKV-specific immune responses might induce more allograft damage due to direct and indirect effects [26].

It is noteworthy that the urine cytology is a very convenient and useful method for diagnosis of PVAN and predicting viremia, especially for the developing countries where PCR may not be easily available. Our previous study, which included 338 recipients with renal transplant biopsies showed that using decoy cells without quantitation had a sensitivity of 95.8% and a specificity of 83.1% for PVAN. If the amount of decoy cells used as positive diagnostic standard was increased, the specificity for prediction of viruia, viremia, and PVAN was increased too. If the amount of decoy cells used as positive diagnostic standard was increased to more than 20 per 10 HPF, the specificity for prediction of viruia, viremia, and PVAN was 100%, 99.7%, and 99.7%, respectively [13].

In summary, we present data suggesting that the extent of interstitial inflammation influences short and long-term graft outcomes in patients with PVAN. Stage of PVAN, viral reduction time, and viral clearance also can be used as prognostic markers in PVAN.

## References

[1] Purighalla R, Shapiro R, McCauley J, Randhawa P. BK virus infection in a kidney allograft diagnosed by needle biopsy. Am J Kidney Dis. 1995; 26:671–673. 7573026 10.1016/0272-6386(95)90608-8

[2] Pham PT, Schaenman J, Pham PC. BK virus infection following kidney transplantation: an overview of risk factors, screening strategies, and therapeutic interventions. Curr Opin Organ Transplant. 2014; 19:401–412. 10.1097/MOT.0000000000000101 25010062

[3] Hirsch HH, Brennan DC, Drachenberg CB, Ginevri F, Gordon J, Limaye AP, et al Polyomavirus associated nephropathy in renal transplantation: interdisciplinary analyses and recommendations. Transplantation. 2005; 79:1277–1286. 15912088 10.1097/01.tp.0000156165.83160.09

[4] Chung BH, Hong YA, Kim HG, Sun IO, Choi SR, Park HS, et a Clinical usefulness of BK virus plasma quantitative PCR to prevent BK virus associated nephropathy. Transpl Int. 2012; 25:687–695. 10.1111/j.1432-2277.2012.01480.x 22509924

[5] Huang G, Wang CX, Zhang L, Fei JG, Deng SX, Qiu J, et al Monitoring of polyomavirus BK replication and impact of preemptive immunosuppression reduction in renal-transplant recipients in China: a 5-year single-center analysis. Diagn Microbiol Infect Dis. 2015; 81:21–26. 10.1016/j.diagmicrobio.2014.09.024 25445121

[6] Kuten SA, Patel SJ, Knight RJ, Gaber LW, DeVos JM, Gaber AO. Observations on the use of cidofovir for BK virus infection in renal transplantation. Transpl Infect Dis. 2014; 16:975–983. 10.1111/tid.12313 25412701

[7] Wadei HM, Rule AD, Lewin M, Mahale AS, Khamash HA, Schwab TR, et al Kidney transplant function and histological clearance of virus following diagnosis of polyomavirus-associated nephropathy (PVAN). Am J Transplant. 2006; 6:1025–1032. 16611340 10.1111/j.1600-6143.2006.01296.x

[8] Gaber LW, Egidi MF, Stratta RJ, Lo A, Moore LW, Gaber AO. Clinical utility of histological features of polyomavirus allograft nephropathy. Transplantation.2006; 82:196–204. 16858282 10.1097/01.tp.0000226176.87700.a4

[9] Bueghrig CK, Lager DJ, Stegall MD, Kreps MA, Kremers WK, Gloor JM, et al Influence of surveillance renal allograft biopsy on diagnosis and prognosis of polyomavirus nephropathy. Kidney Int. 2003; 64:665–673. 12846764 10.1046/j.1523-1755.2003.00103.x

[10] Drachenberg CB, Papadimitriou JC, Hirsch HH, Wali R, Crowder C, Nogueira J, et al Histological patterns of polyomavirus nephropathy:correlation with graft outcome and viral load. Am J Transplant. 2004; 4: 2082–2092. 15575913 10.1046/j.1600-6143.2004.00603.x

[11] Drachenberg CB, Papadimitriou JC, Ramos E. Histologic versus molecular diagnosis of BK polyomavirus-associated nephropathy: a shifting paradigm? Clin J Am Soc Nephrol. 2006; 1:374–379. 17699234 10.2215/CJN.02021205

[12] Huang G, Chen LZ, Qiu J, Wang CX, Fei JG, Deng SX, et al Prospective study of polyomavirus BK replication and nephropathy in renal transplant recipients in China: a single-center analysis of incidence, reduction in immunosuppression and clinical course. Clinical transplantation. 2010; 24: 599–609. 10.1111/j.1399-0012.2009.01141.x 19925472

[13] Huang G, Chen W-F, Wang C-X, Fei JG, Deng SX, Qiu J, et al Non–invasive tool for the diagnosis of polyomavirus BK associated nephropathy in renal transplant recipients. Diagn Microbiol Infect Dis. 2013; 75:292–297. 10.1016/j.diagmicrobio.2012.11.012 23276771

[14] Drachenberg RC, Drachenberg CB, Papadimitriou JC, Ramos E, Fink JC, Wali R, et al Morphological spectrum of polyoma virus disease in renal allografts: diagnostic accuracy of urine cytology. Am J Transplant. 2001; 1:373–381. 12099383

[15] Pappo O, Demetris AJ, Raikow RB, Randhawa PS. Human polyoma virus infection of renal allografts: histopathologic diagnosis, clinical significance, and literature review. Mod Pathol. 1996; 9: 105–109. 8657714

[16] Hirsch HH, Randhawa P; AST Infectious Diseases Community of Practice. BK polyomavirus in solid organ transplantation. Am J Transplant. 2013; Suppl 4:179–188. 10.1111/ajt.12110 23465010

[17] Racusen LC, Solez K, Colvin RB, Bonsib SM, Castro MC, Cavallo T, et al The Banff 97 working classification of renal allograft pathology. Kidney Int. 1999; 55: 713–723. 9987096 10.1046/j.1523-1755.1999.00299.x

[18] Schwarz A, Linnenweber-Held S, Heim A, Bröcker V, Rieck D, Framke T, et al Factors influencing viral clearing and renal function during polyomavirus BK-associated nephropathy after renal transplantation. Transplantation. 2012; 94:396–402. 10.1097/TP.0b013e31825a505d 22836134

[19] Masutani K, Shapiro R, Basu A, Tan H, Wijkstrom M, Randhawa P. The Banff 2009 Working Proposal for polyomavirus nephropathy: a critical evaluation of its utility as a determinant of clinical outcome. Am J Transplant. 2012; 12:907–918. 10.1111/j.1600-6143.2012.03993.x 22390378 PMC3319333

[20] Azzi A, Hirsch HH, Basso S, Fontana I, Cioni M, et al Prospective monitoring of polyomavirus BK replication and impact of pre-emptive intervention in pediatric kidney recipients. Am J Transplant. 2007; 7: 2727–2735. 17908275 10.1111/j.1600-6143.2007.01984.x

[21] Almeras C, Foulongne V, Garrigue V, Szwarc I, Vetromile F, Segondy M, et al Does reduction in immunosuppression in viremic patients prevent BK virus nephropathy in de novo renal transplant recipients? A prospective study. Transplantation. 2008; 85: 1099–1104. 10.1097/TP.0b013e31816a33d4 18431228

[22] Brennan DC, Agha I, Bohl DL, Schnitzler MA, Hardinger KL, Lockwood M, et al Incidence of BK with tacrolimus versus cyclosporine and impact of preemptive immunosuppression reduction. Am J Transplant. 2005; 5: 582–594. 15707414 10.1111/j.1600-6143.2005.00742.x

[23] Weiss AS, Gralla J, Chan L, Klem P, Wiseman AC. Aggressive immunosuppression minimization reduces graft loss following diagnosis of BK virus-associated nephropathy: A comparison of two reduction strategies. Clin J Am Soc Nephrol. 2008; 3: 1812–1819. 10.2215/CJN.05691207 18650404 PMC2572268

[24] Sellarés J, de Freitas DG, Mengel M, Sis B, Hidalgo LG, Matas AJ, et al Inflammation lesions in kidney transplant biopsies: association with survival is due to the underlying diseases. Am J Transplant. 2011; 11:489–499. 10.1111/j.1600-6143.2010.03415.x 21342447

[25] Funk GA, Gosert R, Comoli P, Ginevri F, Hirsch HH. Polyomavirus BK replication dynamics in vivo and in silico to predict cytopathology and viral clearance in kidney transplants. Am J Transplant. 2008; 8:2368–2377. 10.1111/j.1600-6143.2008.02402.x 18925904

[26] Helantera I, Egli A, Koskinen P, Lautenschlager I, Hirsch HH. Viral impact on long-term kidney graft function. Infect Dis Clin North Am. 2010; 24: 339–371. 10.1016/j.idc.2010.02.003 20466274

